# Vitamin D: Effect on Haematopoiesis and Immune System and Clinical Applications

**DOI:** 10.3390/ijms19092663

**Published:** 2018-09-08

**Authors:** Mayte Medrano, Estrella Carrillo-Cruz, Isabel Montero, Jose A Perez-Simon

**Affiliations:** Department of Hematology, University Hospital Virgen del Rocio, Instituto de Biomedicina de Sevilla (IBIS/CSIC/CIBERONC), Universidad de Sevilla, 41013 Sevilla, Spain; mayte_md26@hotmail.com (M.M.); estrellacarrillocruz@gmail.com (E.C.-C.); icas3760@hotmail.com (I.M.)

**Keywords:** vitamin D, haematopoiesis, leukaemia, myelodysplastic syndrome, allogeneic stem cell transplantation

## Abstract

Vitamin D is a steroid-like hormone which acts by binding to vitamin D receptor (VDR). It plays a main role in the calcium homeostasis and metabolism. In addition, vitamin D display other important effects called “non-classical actions.” Among them, vitamin D regulates immune cells function and hematopoietic cells differentiation and proliferation. Based on these effects, it is currently being evaluated for the treatment of hematologic malignancies. In addition, vitamin D levels have been correlated with patients’ outcome after allogeneic stem cell transplantation, where it might regulate immune response and, accordingly, might influence the risk of graft-versus-host disease. Here, we present recent advances regarding its clinical applications both in the treatment of hematologic malignancies and in the transplant setting.

## 1. Vitamin D: Metabolism and Mechanisms of Action

### 1.1. Production and Metabolism of Vitamin D

Vitamin D (vit D) is a fat-soluble steroid synthesized in several steps which ends with the formation of vit D3 or calcitriol, the most active form of vit D. It is known for its role in the regulation of calcium and phosphorus levels as well as bone mineralization. The term vitamin D is imprecise and groups together different components that are part of this family of steroid hormones. Firstly, pre-D3 is produced in the skin from 7-dehydrocholesterol after exposure to ultraviolet irradiation. Besides, this irradiation also produces lumisterol and tachysterol from pre-D3. The synthesis of lumisterol is a reversible process and can be converted back to pre-D, which binds to Vitamin D-binding protein (DBP) and is subsequently removed from the skin. Pre-D3 has to be hydroxylated twice to became fully activated. The first hydroxylation occurs in the liver and, to a lesser extent, in other tissues by 25-hydroxylase (CYP2R1) to produce 25(OH)D. This is the principal circulating form of vit D and provides a clinically useful marker for vit D status. In the kidney, the enzyme CYP27B1 hydroxylates 25(OH)D, which is then converted into 1,25(OH)D. This process is stimulated by parathyroid hormone and inhibited by calcium, phosphate and fibroblast growth factor 23 (FGF-23). CYP27B1 can be also found in other tissues including epithelial cells, immune cells and parathyroid glands. The extrarenal production of 1,25(OH)D is under a different control, mainly by cytokines such as tumour necrosis factor α (TNFα) and interferon γ (IFNγ). In the kidney, the catalytic enzyme CYP24A1 is the responsible for the inactivation of 1,25(OH)D producing 24,25(OH)D [1]. Several oxidative reactions after 24-hydroxilation and conjugation with glucuronic acid generate some compounds that are excreted through the bile.

The balance between 1α-hydroxylase and 24-hydroxylase activities is regulated by calcitriol, calcium and phosphate serum levels. Parathyroid hormone (PTH) stimulates the synthesis of the 1α-hydroxylase under low serum calcium conditions or low levels of vitamin D, resulting in the increase of 1,25(OH)D activation. PTH also inhibits 24-hydroxylase and can induce osteoclast and osteocytes synthesis of the FGF-23, which acts by reducing the expression of renal sodium-phosphate transporters and regulates vitamin D homeostasis by suppressing renal expression of 1α-hydroxylase and inducing 24-hydroxylase, thus reducing serum calcitriol levels and subsequently serum calcium under hyperphosphatemia conditions [2].

**Figure 1:**
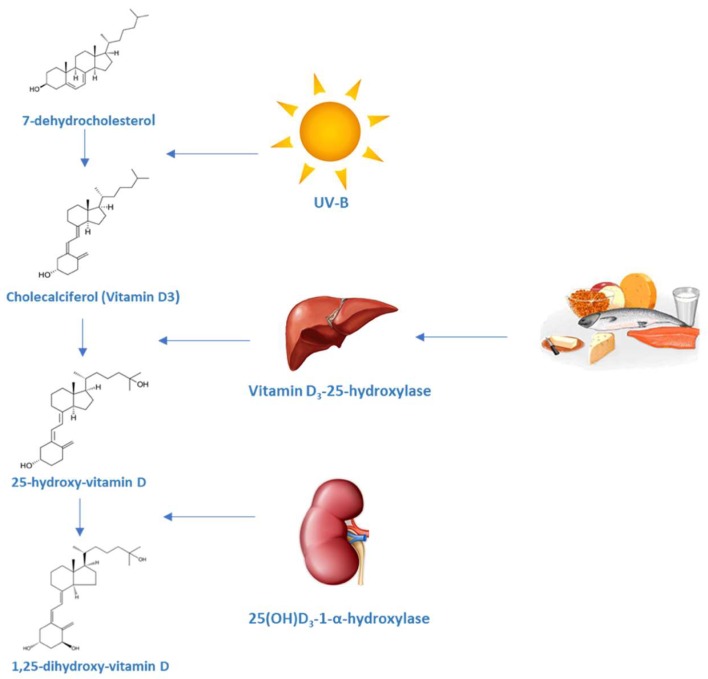
Synthesis of vitamin D and the vitamin D metabolic pathway. The vitamin D metabolites are transported in blood bound primarily to vitamin D binding protein (DBP) (85–88%) and albumin (12–15%).

After ultra violet (UV) exposure, maximum levels of vit D are achieved. From that moment, UV irradiation further converts pre-D to lumisterol and tachysterol preventing higher levels to be reached (Figure 1) [3,4]. Also, the amount of melanin in the epidermis can modify the effectiveness of the sunlight in producing pre-D3 [5].

### 1.2. Vitamin D Receptor (VDR)

Vit D operates via binding vitamin D receptor (VDR). Vit D-VDR heterodimerize with retinoid-X-receptor (RXR) within the cell nucleus where it binds vitamin D responsive elements (VDRE) to serve as a transcription factor for numerous target genes. VDR/RXR complexes can attract co-activators or co-repressors to induce or repress gene transcription, depending on the target gene. Steroid receptor coactivator (SRC) and vitamin D receptor interacting protein (DRIP) complex have been identified as coactivators [6]. SRC coactivators recruit histone acetyl transferases (HAT) to the gene promoting transcription. In addition to acetylation, histone methylation also occurs. Recent studies have shown that methyltransferases may also play a role in VDR-mediated transcription [7]. The DRIP complex acts as a bridge between VDRE and the initiation complex (TATA box, RNA polymerase II and other proteins) to facilitate transcription. Co-repressors of VDR function act in the absence of ligand or in the presence of antagonists. The most studied corepressors for VDR are the nuclear receptor corepressor (NCoR) and the silencing mediator for retinoid or thyroid-hormone receptors (SMRT) [8,9].

1,25(OH)D can also inhibit gene transcription via VDR or inhibiting directly other transcription factors. VDR can also functions un-liganded to 1,25(OH)D. As an example, it modulates mammalian hair cycling in keratinocytes by regulating genes such as *CASP14*, *S100A8*, *SOSTDC1* [10].

The VDR has a very short N-terminal domain when compared to other nuclear hormone receptors. The human VDR has two potential starting sites. A common polymorphism (FokI) alters the first ATG start site to ACG when contains the C genotype (formerly F) instead of T (formerly f). Individuals with C genotype begin translation three codons downstream resulting in a VDR protein three amino acids shorter (424 instead of 427 aa). It has been demonstrated in transfection experiments that the short isoform produces a more potent immune response as it resulted in a higher nuclear factor κB (NF-κB)- and nuclear factor of activated T-cells (NFAT)-driven transcription and a higher IL-12 expression in dendritic cells and monocytes [11]. This polymorphism has also been correlated with a reduced bone [12]. The DNA binding domain is comprised of two zinc fingers. The proximal (N-terminal) zinc finger is a specific site to bind to the VDREs whereas the second zinc finger serves for heterodimerization to the retinoid X receptor (RXR). The second half of the molecule is the ligand binding domain, where it binds 1,25(OH)D, which also contains regions required for heterodimerization to RXR. The major activation domain, AF-2, is located at the C-terminal end, which is critical for the binding to coactivators [6].

## 2. Effect of Vitamin D on the Immune System

In addition to its effect on calcium metabolism, vit D plays other important physiological roles. These effects are called “non-classical actions” and were identified 30 years ago, when receptors for 1,25-dihydroxyvitamin D3 (1,25(OH)_2_D3) were detected in several cell lines [13,14,15]. In fact, VDR is present in most cells types, which explains its wide range of effects [16]. As previously mentioned, vit D is primarily involved in calcium and phosphate homeostasis. Besides, vit D has other functions, such as regulation of hormone secretion. More specifically, 1,25(OH)D stimulates insulin and thyroid-stimulating hormone (TSH) secretion. Pancreatic β cells have CYP27B1, VDR and calbindin-D. Studies with calbindin-D null mice suggest that it modulates depolarization-stimulated insulin release [17,18].

Vitamin D is also implicated in the regulation of proliferation and differentiation of cells. As an example, vit D is involved in normal breast development and in hepatic cell growth. 1,25(OH)D collaborates in the maturation of type II epithelial pneumocytes by increasing phospholipid production and surfactant release and stimulates the innate immune response in bronchial epithelial cells [19]. Vit D stimulates calcium uptake by cardiac muscle cell [20], which is necessary to the contractility. It has been reported that vit D deficiency is associated with increased risk of myocardial infarction in men [21]. Vit D is also essential for skeletal muscle function. Therefore, vit D deficiency produce proximal muscle weakness [22].

Within the spectrum of non-classical effects of vitamin D, actions on cells of the immune system are included [23,24]. In patients with granulomatous diseases such as sarcoidosis, high levels of 1,25(OH)_2_D3 and hypercalcemia are observed. An increase in the enzyme 25-hydroxyvitamin D-1-α-hydroxylase (1α-hydroxylase) is also observed. Unlike normal subjects, in whom the activity of this enzyme is located in the kidney, in patients with sarcoidosis, activity is also observed in macrophages [25,26,27,28]. The deregulation of 1,25(OH)_2_D3 is not restricted to sarcoidosis but is a common feature in many granulomatous diseases [29]. The precise nature of the interaction between vitamin D and the immune system took many years to identify and there are still many questions about this interaction.

### 2.1. Vitamin D and Innate Immunity

#### 2.1.1. Macrophages, Vitamin D and Cathelicidin

Macrophages and monocytes play a key role in the initiation of non-specific responses to pathogenic organisms or tissue damage. This role consists in phagocytizing pathogens or cellular debris and then eliminating or assimilating the material.

For many years it was thought that the most important action of vitamin D on macrophages was due to its ability to stimulate the differentiation of monocytic precursors to mature macrophages [23,30,31,32]. This concept was supported by observations that showed different expression of the vitamin D receptor (VDR) and α-1 hydroxylase in the different stages of differentiation of macrophages. Some studies show that human macrophages are able to synthesize 1,25(OH)_2_D3 upon exposure to IFNγ [33,34]. The confirmation of this effect on monocytes was obtained by Modlin et al. who described genes involved in innate immunity regulation which are specifically modulated in monocytes by *Mycobacterium tuberculosis*. VDR and the gene coding for 1-α-hydroxylase (CYP27B1) are induced through the “toll like” 2/1 receptor pathway (TLR 2/1). The interaction of TLR 2/1 with the precursor of vitamin D (25(OH)D3) stimulates the expression of the antibacterial protein cathelicidin [35]. Regulation of the transcription of cathelicidin by binding VDR to 25(OH)D3 is possible because its promoter gene contains a functional VDRE. This element is only present in the most developed primates, which is suggestive that the regulation of vitamin D in this facet of innate immunity is a recent event of evolutionary development [36,37]. The precise mechanism by which TLR activation induces the expression of VDR and 1-α-hydroxylase is not clear. The analysis of the events involved in the transcriptional regulation of CYP27 B1 suggests that the interaction with TLR involves the JAK/STAT pathway, the MAP kinases and NF-κB and that all of this occurs in synergy with the induction of CYP27B1 by IFNγ [38]. Other studies propose that the induction of CYP27B1 by TLR2/1 occurs indirectly through IL-15 and IL17A. The enzyme 24-hydroxylase, whose function is to inactivate 1,25(OH)_2_D3, is encoded by a gene (CYP24) that is induced by 25(OH)D3 after activation by TLR2/1 in monocytes [38,39].

In summary, vitamin D is a potent stimulator of the mechanisms associated with the elimination of pathogens and the appearance of this system in primates (including early Homo sapiens), exposed to abundant amounts of sunlight, was an evolutionary advantage. In fact, it is a system that has key control mechanisms; not only has a catabolic enzyme, 24-hydroxylase, which attenuates the responses to 1,25(OH)_2_D3 but also feedback mechanisms. In this regard, 1,25(OH)_2_D3 is a potent down-regulator of TLR2 and TLR4 in monocytes, decreasing inflammatory responses. Hence, using CYP24 and TLR regulatory mechanisms, vitamin D helps to promote innate immune responses by preventing tissue damage associated with excessive inflammation [40,41].

#### 2.1.2. Dendritic Cells and Antigenic Presentation

Dendritic cells (DC) are responsible for the presentation of antigens, resulting from the elimination of pathogens and tissue debris, to cells belonging to adaptive immunity.

Myeloid dendritic cells (M-DCs) produce high levels of IL-12, whereas high levels of IFN are secreted by plasmacytoid dendritic cells (P-DCs) with distinct effects on activation and differentiation of T-cell [42].

Dendritic cells participate in immune responses producing both immunogenic and tolerogenic phenotype. Immature DCs, by the expression of inhibitory receptors, induce anergy among CD4+ cells and elicit generation of IL-10 producing T regs cells [43]. The tolerogenic activity of immature DC may also be related to the expression of endocytic manose receptors (MR), which can deliver negative signals to T-cells. MR levels are up-regulated by anti-inflammatory molecules such as corticosteroids, vit D, Th2 cytokines (IL-4 and IL-3) and are down-regulated by proinflammatory stimuli (IFN-α). At the same time, Tregs are capable of maintaining the tolerogenic state by inhibiting myeloid DC maturation, reducing their antigen-presenting function and decreasing IL-12 secretion [44].

The expression of VDR in purified dendritic cells was reported for the first time in 1987 [45]. Studies carried out subsequently purifying skin dendritic cells (Langerhans cells) showed that 1,25(OH)_2_D3 could attenuate the effect of antigen presentation [46]. However, the role of vitamin D and its metabolites was not elucidated until the advent of dendritic cell models derived from monocytes.

In the year 2000, Gauzzi et al. showed that 1,25(OH)_2_D3 and its synthetic analogues inhibited the maturation of dendritic cells derived from monocytes, suppressing their ability to present antigens to T cells, suggesting vitamin D could promote tolerance [47,48,49]. This concept was evaluated in pancreatic islet transplant models in which a lower rejection rate was observed in mice treated with vitamin D [50]. This response seems to be due to a decreased maturation of DC and a concomitant involvement of suppressor T lymphocytes or regulatory T cells [51].

Overall, vit D induces DCs tolerogenicity due to the capacity to inhibit differentiation, maturation, costimulatory molecule expression and IL-12 production, leading to decreased allostimulatory capacity while enhancing IL-10 secretion which favours the induction of regulatory T cells. Vit D not only inhibits DC differentiation and maturation but also transform differentiated IFN-DC into a more immature stage [47].

1,25(OH)_2_D3 preferentially affects myeloid dendritic cells. Although some studies suggest an apparent insensitivity of plasmacytoid dendritic cells to 1,25(OH)_2_D3, it is possible that the local synthesis of vitamin D by these cells could participate in the mechanism of tolerogenicity through the VDR receptor expressed in T cells [52,53].

### 2.2. Vitamin D and Adaptive Immunity

#### 2.2.1. Vitamin D and T Lymphocytes

Non-activated T cells express undetectable levels of VDR, while receptor expression increases upon T cell activation. Overall, vit D could limit the potential tissue damage associated with Th1 immune responses. However, the validity of this generalization was questioned in mouse models in which vit D was able to inhibit the cytokines associated with both Th1 (INFγ) and Th2 (IL-4) [54,55,56,57,58]. In addition, it is interesting to note that in animal models of inflammatory bowel disease, the treatment with vit D reduces the expression of IL-17 [59,60]. Hence, it is possible that vit D exerts its effects on inflammation and autoimmune disease also through the regulation of Th17 cells. Finally, vitamin D also affect regulatory T cells. In this regard, in 2002, it was shown that 1,25(OH)_2_D3 in conjunction with steroids stimulated the production of IL-10 by the CD4+ CD25+ regulatory T cells [51,61]. Other studies indicate that vitamin D produces a preferential T-regulatory differentiation [62,63]. Accordingly, vitamin D stimulates the secretion of IL-10 and TLR9 by regulatory T cells, which connects immune and adaptive responses [64].

In contrast to the wide effect on CD4+ T cells, CD8+ T cells show a poor response to 1,25(OH)_2_D3 [65,66,67,68]. Despite their significant expression of VDR, 1,25(OH)_2_D3 does not have a significant impact on CD8+ lymphocytes, as shown in animal models of autoimmune encephalomyelitis [69,70].

In addition to its effect on certain T cell populations, recent studies show that vitamin D may also have an effect on the phenomenon of “homing” or tropism of T cells by specific tissues [71]. Some studies suggest that vitamin D inhibits the migration of T cells to lymph nodes. It might also have an effect on T cell homing into the skin by upregulating the cytokine receptor (CCR10), whose ligand CCL27 is expressed by epidermal keratinocytes. This effects on the phenomenon of “homing” is produced by both 25(OH)D3 and by 1,25(OH)_2_D3 and several authors suggest that both dendritic cells and T lymphocytes are the source of 1α hydroxylase activity [72]. By contrast, vitamin D exert a negative effect on cytokines and their receptors in the gastrointestinal tract [73].

#### 2.2.2. Vitamin D and B Lymphocytes

In addition to the classical function of B lymphocytes as precursors of plasma cells that produce antibodies and, therefore, as a cellular subtype in which humoral immunity rests, populations of B lymphocytes whose main function is the production of IL-10 have been described that could correspond to regulatory B subpopulations. Some animal models of autoimmune diseases have revealed that the regulatory B cells produce IL-10 and can suppress inflammatory processes. IL-10 derived from B cells seems to be important for the generation and maintenance of regulatory T cells. In models in which the maturation of B cells is blocked, there is a reduced capacity for the induction of tolerance and this inability is associated with inadequate potential for the generation of FoxP3+ regulatory T cells. These findings are corroborated by murine models of arthritis in which the deficiency of IL-10 derived from B cells exacerbates the disease and correlates with low levels of regulatory T cells, which are restored with the transfer of IL-10 producing B cells [74,75,76,77,78,79,80,81]. Deficits in the function and frequency of regulatory B cells have been reported in multiple sclerosis and systemic lupus erythematosus [82,83,84,85].

Like T cells, active B cells express VDR. As shown in experimental studies, B cells at rest have low but detectable levels of mRNA for VDR [86,87]. After incubation with anti CD40/IL-21, an increase in VDR expression is observed. The addition of 1,25(OH)_2_D3 increases the expression of VDR mRNA. VDR is functional in activated B cells [85] while the mRNA for CYP24A1 is not detectable in B cells at rest [88]. Activated B cells are therefore able to metabolize vitamin D and respond to its active component.

Initial studies indicated that 1,25(OH)_2_D3 could regulate the production of B cells and the secretion of immunoglobulins [89]. Studies in which purified B cells are cultivated in the presence of vitamin D show a decrease in the percentage and absolute number of plasma cells as well as IgA, IgG and IgM levels, the results being contradictory with IgE [90,91,92,93,94,95]. The formation of memory B lymphocytes is also inhibited by 1,25(OH)_2_D3. Therefore, the effects of vitamin D on B cells could be summarized as a decrease in the “pool” of memory B cells and inhibition of the generation of plasma cells, with the consequent decrease in the secretion of antibodies. On naïve B cells, constitutively expressing VDR and CYP27B1, the response to vitamin D consists in an increase of the expression of VDR, of 25(OH)D3 and of the degrading enzyme of 1,25(OH)_2_D3 CYP24A1. After activation, the expression of VDR and CYP27B1 does increase, which also results in an increase of the level of 1,25(OH)_2_D3 and stimulates the negative feedback mechanism through the increase of CYP24A1 [85]. The participation of regulatory B cells in the immune homeostasis exerted by vitamin D seems to be confirmed in murine models of autoimmune diseases in which a functioning pathway mediated by IL-10 is required to guarantee the effect of 1,25(OH)_2_D3 on the disease [96,97].

### 2.3. Genetic Fingerprint of Vitamin D in the Immune System

Deep genomic analysis has allowed to define a new perspective on vitamin D and its function in humans. In macrophages, the description of increased levels of VDR and 1α hydroxylase (CYP27B1) after a pathogenic challenge underscored the importance of the intracrine system of vitamin D as a mediator of the immune response [34,98]. It is now known that macrophages and dendritic cells are capable of responding to 25(OH)D3, the largest circulating metabolite of vitamin D, which provides a link between these cells and vitamin D in humans. The identification of hundreds of target genes for 1,25(OH)_2_D3 in immune cells has also provided a new perspective on the role of vitamin D in the adaptive immune system [33].

The first study based on genome assays focused on vitamin D was published by John White and colleagues at McGill University in Montreal and used a combination of DNA arrays on genes regulated by 1,25(OH)_2_D3 and strategies in silico [26,27,33,99,100,101,102,103]. The deep analysis of the target sequences that are capable of binding to VDR reveals response elements (VDRE) that are located next to genes that promote antibacterial proteins such as cathelicidin (CAMP) and β defensin 2 (DEFB4). Only CAMP seems to be induced transcriptionally in monocytes [104]. The underlying mechanism for the differential regulation of CAMP and DEFB4 by 1,25(OH)_2_D3 was determined in subsequent studies. The first one described the increased expression in monocytes of DEFB4 after treatment with 1,25(OH)_2_D3 and IL-1. This required the cooperative occupation of VDRE by NF-κB and, on the other hand, the binding of VDRE with the DEFB4 promoter gene [104].

The importance of NF-κB and VDR as co-inductors of the transcription of β defensin 2 (DEFB4) was subsequently reinforced in studies focused on the nucleotide-binding oligomerization domain-containing protein 2 (NOD2) protein [105]. Cells treated with 1,25(OH)_2_D3 and the NOD ligand multidrug resistance (MDR), derived from microorganisms, show a potent induction of DEFF4 dependent on NF-κB [36,37]. However, the induction of CAMP is primarily dependent on the binding of VDR to the VDRE promoter. The VDRE element initially identified as the CAMP promoter appears to be specific to human and subhuman primates [106]. The acquisition of a VDRE element for the CAMP gene seems to have occurred due to the introduction of a nuclear element (SINE) that put CAMP under the control of the VDR receptor. This specific adaptation of primates has been conserved in humans and in primates of the Old and New World, suggesting that CAMP’s regulatory transcriptional mechanism for vitamin D confers biological advantages. It is assumed that this mechanism could be potently activated by the relatively high levels of 25(OH)D3 and 1,25(OH)_2_D3 that are characteristic of non-human primates.

Recognition and response to pathogens involves the identification of molecular patterns associated with pathogen surveillance (PAMPs) through pattern recognition receptors (PRRs), including the extensive family of “Toll like” receptors (TLR), noncatalytic transmembrane proteins that interact with specific PAMPs [35]. In genomic studies on models of tuberculous infection, the TLR 2/1 stimulus induces the expression of CY27B1 and VDR, suggesting that the endocrine system of vitamin D is involved in the macrophagic response to *Mycobacterium tuberculosis*. The macrophages treated with ligand TLR1/2 are reactive to the 1,25(OH)_2_D3 and 25(OH)D3 forms of vitamin D, confirming the functional efficacy of the intracrine model.

The stimulation of TLR1/2 by *Mycobacterium tuberculosis* also produces the induction of the catabolic enzyme of vitamin D (CYP24A1) and the antibacterial protein CAMP. The expression of the other antibacterial protein DEFB4 is a result of the cooperative action between the TLR1/2, Il-1, NOD2 MDP pathway. Antibacterial proteins such as CAMP and DEFB4 play a crucial role in bacterial intracellular death mediated by vitamin D. Monocytes treated with increasing concentrations of the CAMP peptide show a dose-dependent reduction of internalization of *Mycobacterium tuberculosis* and a similar inhibition in macrophages occurs in presence of 25(OH)D3, this effect being interrupted by the VDR antagonists [37]. Vitamin D and its analogues are capable of promoting autophagy, this induction being very important to provoke antibacterial responses through vitamin D in the tuberculous infection [107].

The intracrine synthesis of 1,25(OH)_2_D3 seems to regulate the expression of another antibacterial protein, hepcidin (HAMP) [107,108]. The major function attributed to HAMP seems to be the suppression of the membrane protein ferroportin, the only intracellular iron exporter. This link in cells such as enterocytes, hepatocytes and monocytes play a key role in the so-called chronic process anaemia [107]. Iron restriction from the circulation provides an important host response to systemic infection, although for pathogens such as *Mycobacterium tuberculosis*, that evades immune surveillance at the intracellular level, iron accumulation might favour the growth of intracellular pathogens. Vitamin D in its forms 25(OH)D3 and 1,25(OH)_2_D3 suppress the transcription of HAMP in monocytes and hepatocytes, which leads to the release of the blockade to ferroportin dependent on HAMP, favouring the transport of iron and decreasing its intracellular concentration [109].

Neutrophils express VDR but, unlike monocytes and macrophages, they do not seem to express a functional 1α-hydroxylase enzyme and are not subject to an intracrine activation of the vitamin D system [110].

Dendritic cells, which belong to the same hematopoietic lineage of monocytes and macrophages, express VDR and CYP27B1 and exhibit an active intracrine system of vitamin D [45,110,111]. As discussed above, dendritic cells use a paracrine system of vitamin D, in which differentiation into antigen-presenting cells implies an increase in CYP27 B1 and, paradoxically, a decrease in VDR expression [112]. Therefore, immature cells express VDR and respond to 1,25(OH)_2_D3 produced by mature cells, with low expression of VDR. Such mechanism pursues the maturation of the dendritic cell and the promotion of T activation and prevents the over-elaboration of the immune response.

In mice, CD8 cells express the activating enzyme of vitamin D, 1-α-hydroxylase [67]. However, in experimental models of autoimmune diseases mediated by CD8 lymphocytes, vitamin D does not seem to play any role. CD8αα, a variant of CD8 T cells and vitamin D are tied and play a role in the suppression of gastrointestinal inflammation [113].

### 2.4. Vitamin D Levels and Immune Function

Most of the deep genomic analyses that explore the immunomodulatory effects of vitamin D in vitro have focused on the use of 1,25(OH)_2_D3 or its synthetic analogues. However, the induction by pathogens of an intracrine system such as that of monocytes/macrophages suggests that in vivo regulation is independent of 1,25(OH)_2_D3. Probably, this system is initially driven by the local activation of 25(OH)D3, the predominant circulating form of vitamin D.

Epidemiological studies have shown that insufficient levels of vitamin D (serum levels below 30 ng/mL) are associated with an increased risk of tuberculosis [114,115,116,117]. Clinical trials with vitamin D supplements added to conventional antibiotics have shown variable success. When 10,000 IU of vitamin D were used daily, serum vitamin D levels increased in tuberculous patients but it did not improve the efficacy of the treatment as compared to patients included in the placebo arm. However, in a specific subgroup of patients with a single Taq1 nucleotide polymorphism in the VDR gene, this reduction was demonstrated, suggesting that genetic factors may influence the immune response to vitamin D supplementation [118].

The link between vitamin D and infection is not restricted to patients with tuberculosis. Among patients with sepsis, circulating levels of 25(OH)_2_D3 are correlate with serum concentrations of CAMP and also correlates with poor prognosis [119,120]. Low levels of 25(OH)D3 are linked to respiratory infections such as influenza and, in patients with chronic renal failure, are correlated with an increased rate of infection and mortality [121]. The application of deep genomic analysis to assess the impact of serum vitamin D status on immune function is limited [122]. In animal models, deep analysis of immune responses has been attempted [123]. Mice deficient in vitamin D showed a decreased expression of angiogenin 4, an antibacterial protein that acts by minimizing the invasion of tissues by enteric bacteria. This leads to higher levels of bacteria in the colon epithelium [124]. This deregulation is related with tissue inflammation in inflammatory bowel diseases and, accordingly, vitamin D could protect from this inflammation by inducing the antibacterial protein angiogenin 4 [125].

A recent study of almost 34,000 individuals shows that genetic variations in DBP influence on the serum concentrations of the DBP protein which, in turn, are linked to the total serum levels of 25(OH)D3 and 1,25(OH)_2_D3. The genetic variations of the DBP protein could be related to different affinities of 25(OH)D3 with the DBP protein [126,127,128]. The antibacterial responses to 25(OH)D3 are more pronounced with forms of low affinity BPD involving high free levels of 25(OH)D3 [129,130].

## 3. Vitamin D in Haematopoiesis and Hematopoietic Stem Cells

The physiologically active form of vitamin D, 1,25-dihydroxycholecalciferol or 1,25(OH)_2_D3 promote monocytic differentiation of HL60, a human promyelocytic leukaemia cell line [131,132]. It is also well recognized that 1,25(OH)_2_D3 induces normal mononuclear blood cells to differentiate towards the monocyte-macrophage route of maturation [133]. Studies in vitro shown that vitamin D suppresses colony formation of normal human granulocyte macrophage progenitors (CFU-GM) and, by contrast, induces differentiation of colonies into monocyte-macrophages [134]. Experiments with hematopoietic stem cells and leukemic cell lines treated with the active form of vitamin D demonstrate an increase in monocyte/macrophage differentiation and an increase in the number of mature cells that is not evident in mice lacking VDR [135,136,137]. After its binding to the vitamin D analogue, VDR forms a homodimer or binds to the retinoid X receptor (RXR), which proceeds to interact with VDRE originating a transcription signal on several effector RNAs [56,136]. In addition to RXR, VDR can also bind to the retinoic acid receptor (RAR) which promotes the differentiation of mature granulocytes [138]. Experiments with cell cultures suggest that activated RAR and VDR compete for their binding to RXR and the relative balance between the RAR/RXR and VDR/RXR dimers influences the relative activity of granulopoiesis or monopoiesis [139]. In animal models, the lack of VDR and RAR allows the generation of appropriate monocyte and granulocyte colony forming units, with which it is suspected that the primary effect of vitamin D occurs on cytokine signalling and on the final stages of differentiation of these two cell types.

The complexity of the interaction between VDR and RXR has been reflected in recent observations in which it is shown that retinoic acid and vitamin D can potentiate their action mutually, so that VDR stimulation seems to increase in the presence of RAR-binding. RXR [136].

**Figure 2:**
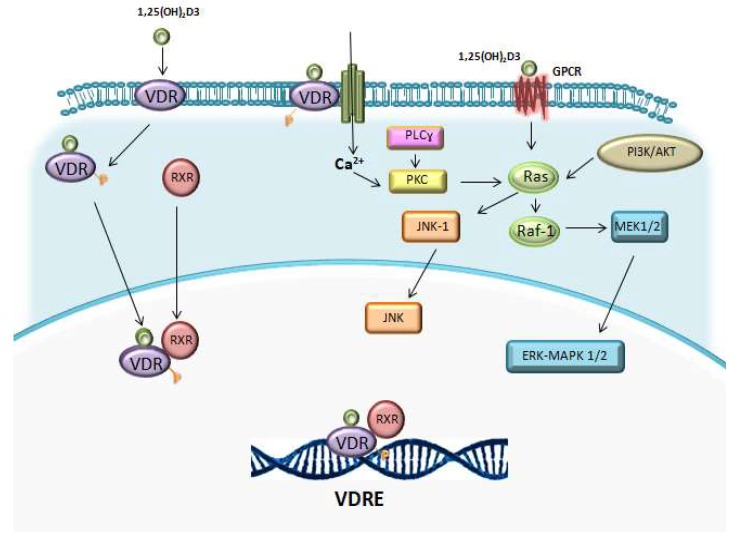
1,25(OH)_2_D3 acts mediating lipid signalling pathways (protein kinase C pathway), phosphatidylinositol-3-kinase (PI3K)-AKT pathway and mitogen activated kinase (MAPK) pathways.

Studies with VDR knockout (KO) mice showed that the lack of VDR does not affect the normal haematopoiesis and mice presented normal relative numbers of red and white blood cells. By contrast, the addition of vitamin D derivatives can influence at later stages of haematopoiesis [56,140]. 1,25(OH)_2_D3 activates certain intracellular signalling pathways, which have been suggested to intersect at a common nodal point, Raf-1, such as: lipid signalling pathways (protein kinase C pathway), the phosphatidylinositol-3-kinase (PI3K)-AKT pathway and mitogen activated kinase (MAPK) pathways (Figure 2) [131,141].

Regarding the lipid signalling pathway, vitamin D can increase the activity of sphingomyelinase and protein kinase C (PKC) [131,141]. The latter can influence on signal transduction through MAPK pathways. In addition, PKC is an important mediator of hematopoietic cell differentiation [142].

Lipid signalling pathways involve sphingomyelinase, whose activity increases in HL-60 cells treated with 1,25(OH)_2_D3, leading to increased ceramide levels and enhancement of vitamin D-induced differentiation [143,144].

1,25(OH)_2_D3 can activate PI3K-AKT pathway, involved in the formation of a VDR/PI3K complex in a signalling pathway that parallels the MAPK pathways, which mediate cell differentiation. LY294002, a PI3K inhibitor, inhibits 1,25(OH)_2_D3-induced monocytic cell marker CD14 and CD11b expression in THP-1 cells, demonstrating the implication of PI3K pathway in promoting differentiation [145,146]. Vitamin D has also been proposed to induce differentiation by disassembling AKT-Raf1 complex, upregulating Raf1 and activating the Raf/MEK/ERK MAPK pathway.

Three different MAPK signalling cascades are implicated in 1,25(OH)_2_D3-induced cell growth arrest and differentiation: Raf-1/MEK/ERK MAKP, JNK/MAPK and p38/MAPK pathways [131].

With regards to lymphopoiesis, Yu et al. demonstrated, using a VDR KO mice lacking vitamin D, that the expression of the vitamin D receptor (VDR) is required for normal thymic development and function of invariant Natural Killer T (iNKT) cells, which are intrinsically defective and lack T-bet expression. In vitro studies showed an inhibitory effect of vitamin D on NK cell development, while promoting myeloid differentiation. However, analysis of CD4 and CD8 T cells and regulatory T cells numbers in the thymus identified no differences between wild type (WT) and VDR KO mice [73,147,148].

VDR KO mice present an extramedullary haematopoiesis because of abnormal bone mineralization [149]. Hematopoietic defects such as anaemia, extramedullary haematopoiesis, thrombocytopenia, myelofibrosis and myelodysplasia was exhibited by children with vitamin D deficiency-associated rickets [150]. 1,25(OH)D3 affects embryonic hematopoietic stem and progenitor cell (HSPC) numbers in vivo and in vitro via VDR-mediated regulation of pro-proliferative responses independent of Ca^2+^ flux [150].

1,25(OH)D3 negatively influences hemogenic endothelial formation independent of VDR activation by antagonizing Hedgehog signalling [151]. Ex vivo treatment with 1,25(OH)D3 increased the proliferation, survival and multi-lineage colony forming activity of CD34+ human umbilical cord blood hematopoietic stem cells (HSCs) [150].

## 4. Clinical Applications

### 4.1. Use of Vitamin D in the Treatment of Hematologic Malignancies

Due to the aforementioned effects on maturation, vitamin D and its analogues have been used as treatment in myeloid neoplasms, particularly myelodysplastic syndromes (MDS) and acute myeloblastic leukaemias (AML). Specific preclinical experiences with HL-60 and other leukemic lines, such as U-937 and THP-1, have shown differentiation and apoptosis of blasts with vitamin D, suggesting that these components, such as all-*trans* retinoic acid (ATRA) in acute promyelocytic leukaemia (APL), reverse the blockade of the differentiation of myeloblasts [131,136,141].

The anti-leukemic activity of vitamin D was described almost three decades ago as Tanaka showed that treatment with a vitamin D analogue, improved survival in leukemic mice [32]. Muto demonstrated that calcitriol can inhibit cell cycle and induces differentiation of leukaemia cells through VDR [152]. Other numerous studies on AML blasts or leukemic cell lines showed that vitamin D induce cell differentiation and growth inhibition [30,31,141,145,153,154,155]. The exact mechanism by which the activation of the vitamin D receptor induces this effect is not completely clear and investigations have revealed complex cross-signals involving P13 kinase, MAPK pathway and probably the upregulation of factors such as p53 [131].

Unfortunately, early preclinical trials using supraphysiological doses that induced differentiation also induced hypercalcemia [136,156]. However, other studies showed that fractionated doses could achieve the same effect on differentiation, maintaining the level of vitamin D in the physiological range [137].

There are several studies in which vitamin D therapy has been used as the single agent in myelodysplastic syndromes (MDS). The first study was carried out in 1985 by Koeffler and colleagues, reporting 18 patients treated with 1,25(OH)_2_D3 with a dose greater than 2 mg/day. Although 8 patients presented minor haematological responses, the response did not persist for more than 12 weeks and hypercalcemia was a common toxicity [156].

In a retrospective study conducted by Hermine et al. [157], it was shown that VDR expression in AML is correlated to prognosis. Accordingly, patients presenting higher VDR expression have an increased survival. Moreover, patients’ prognosis is correlated to the expression of VDR-targeted genes. Patients with higher CAMP expression presented an increased event free survival (EFS) compared to patients with lower levels of CAMP expression.

Other studies with vitamin D analogues failed to demonstrate haematological responses in MDS although tolerance was adequate [158]. Recently, Motomura et al. randomized a series of 30 patients to receive 25(OH)D3 versus supportive treatment. Only one of the 15 patients in the vitamin D group progressed to AML versus seven in the control group [159].

There are several attempts to combine vitamin D with other cytotoxic agents. Siitonen et al. reported a series of 19 patients with MDSs treated with a combination of 13-*cis* retinoic acid, 1,25(OH)_2_D3 (1 mg/day) and valproic acid, used as a histone deacetylase inhibitor [160]. Three patients had haematological response but intolerance was recorded in eight patients, due to 13-*cis* retinoic acid and valproic acid.

In 2008, a study was conducted with 63 patients with MDS with a combination of erythropoietin, 13-*cis* retinoic acid, 1,25(OH)_2_D3 and thioguanine in the presence of blasts. An overall erythroid response of 60% was obtained, reaching 93% in low risk patients [161]. Subsequently, vitamin D analogues were combined with cytotoxic chemotherapy in a series of 53 patients with MDS in whom 13-*cis* retinoic acid (20–40 mg daily) was added to 1,25(OH)_2_D3 (1–1, 5 mg daily) with or without thioguanine. The overall response rate was 60% and 50% of patients achieved transfusional independence [162].

The only study that used vitamin D monotherapy in AML was conducted in the eighties on 5 patients, four of whom presented a transient reduction in the number of blasts and only one described a brief normalization of the spinal study [163]. In 1992, Slapak et al. reported the use of continuous infusion of cytarabine for 21 days (20 mg/m^2^ daily), hydroxyurea (500 mg twice daily) and 1,25(OH)_2_D3 (0.5 mg twice a day) [162]. An overall response rate of 79% was obtained with 45% complete response rate. The toxicity was primarily haematological consisting of neutropenia and thrombocytopenia, only two patients developed asymptomatic hypercalcemia that did not require treatment. These results were considered at least not inferior to those achieved with cytarabine as the sole agent. Ferrero and colleagues carried out another study in 2008 with a similar population of 30 patients (24 AML, 6 MDS) in which they used subcutaneous cytarabine (8 mg/m^2^ twice a day), 1,25(OH)_2_D3 (1 mg daily), 13-*cis* retinoic acid (20–40 mg daily) and thioguanine (40 mg daily). The median survival was 7.5 months, being 16 months for the responders. The toxicity was mainly related to cytopenias [164].

The only study published in the literature comparing combination treatment with vitamin D analogues (1,25(OH)_2_D3 1 μg/day) and chemotherapy (cytarabine 15 mg/m^2^ daily subcutaneously until the blasts were lower 50%) compared to a control group was performed in Sweden on a total of 78 patients, 68 with MDS and 15 with AML. Half of the patients also received 13-*cis* retinoic acid (1 mg/kg daily). The mean survival was 10.5 months, with no significant difference between both groups [165].

Interestingly, vitamin D serum levels have also been correlated to response rate to 5-azacytidine (AZA) among patients with MDS or AML. In this regard, Radujkovic et al. analysed serum levels before starting AZA in 58 patients. Estimated probability of 2-year overall survival in the low versus high vitamin D levels group was 14% versus 40% (*p* < 0.05). In multivariable analysis, adverse cytogenetics and vitamin D levels were independent predictors of survival [166]. Similarly, Lee et al. reported a series of 97 patients diagnosed with AML who received intensive chemotherapy; in this study, a significantly worse outcome was observed among those patients with low vitamin D levels [167]. These data are in contrast to those reported by Pardanani et al. who did not find any relationship between vitamin D levels and prognosis in a series of 409 patients diagnosed with different myeloid neoplasms and MDS [168].

There are also studies evaluating the role of vitamin D in lymphoid malignancies. In this regard, several preclinical studies have demonstrated activity of the vitamin D analogue EB1089 in the multiple myeloma cell line H929. This agent promotes apoptosis and induce cell cycle arrest by downregulation of cyclin-dependent kinases [169,170,171]. There are preclinical studies that show that vitamin D has an inhibitory effect on neoplastic lymphoid cells but to date there have been no studies in humans [172].

A retrospective study performed by Kelly J et al. analysed 183 patients who were enrolled in three SWOG trials and had 25(OH)D serum levels available. There was no association between vitamin D deficiency and clinical response. After a median follow-up of 5.4 years, vitamin D-deficient patients had a significantly worse progression free survival (PFS) (hazard ratio (HR), 2.00; *p* = 0.011) and overall survival (OS) (HR, 3.57; *p* = 0.003) as compared with those with higher levels. Multivariable analysis suggested that lower levels of vitamin D were associated with a higher risk of either progression or death but neither result was significant. In addition, 240 patients enrolled onto the parent PRIMA clinical trial were analysed. After a median follow-up of 6.6 years, vitamin D-deficient patients had significantly lower PFS (HR, 1.66; *p* = 0.013) but not OS (HR, 1.84; *p* = 0.14) as compared with those with higher levels Multivariable analysis confirmed that lower levels of vitamin D were associated with a higher risk of either progression or death [173].

Similar results have been reported by Tracy SI et al. in a series of 642 patients with follicular lymphoma. The authors evaluated whether vitamin D insufficiency was associated with adverse outcomes; with a median follow-up of 59 months, 297 patients (46%) had an event (progression, treatment failure), 78 had died and 42 (6.5%) had a lymphoma-related death. Vitamin D deficiency was associated with inferior EFS at 12 months (OR = 2.05; 95% confidence interval (CI) 1.18–3.54), OS (hazards ratio (HR) = 2.35; 95% CI 1.37–4.02) and lymphoma-specific survival (HR = 2.97; 95% CI 1.52–5.80) for the full cohort [174].

In diffuse large cell lymphoma Hohaus et al. analysed 128 patients. 25(OH)D levels below 20 ng/mL at diagnosis and IPI were independently associated with a worse EFS. Moreover, patients with normalized 25(OH)D levels following supplementation showed a better EFS as compared to those patients with persistent insufficient 25(OH)D levels [175].

### 4.2. Vitamin D as a Modulator of the Immune Response in Allogeneic Transplantation

There is considerable interest in vitamin D analogues for their immunomodulatory effects, which could be considered an effective approach among patients undergoing allogeneic hematopoietic stem cell transplantation (HSCT) to prevent graft-versus-host disease (GVHD). The VDR genes are polymorphic in the human population and this genetic variation in VDR has been investigated in patients undergoing HSCT. Cho and colleagues conducted an analysis of 147 patients. They analysed the polymorphisms for VDR and evaluated the association with the prognosis of the patients. They showed a correlation between the polymorphisms of the anchor site of the restriction enzyme Taq1 and survival, so that heterozygotes (who have at least one copy of the C allele) had a better overall and disease-free survival than homozygous TT. The functional significance of this allelic variation is unknown and no direct association with a higher or lower VDR activity was reported. This study also found that recipients with two copies for the “A” allele, related to polymorphisms for the Apal anchor site, had a lower risk of acute GVHD and infections [176]. These polymorphisms have been related to VDR activity so that homozygosity for the “a” allele translates into greater activity [177].

Middleton and colleagues studied a cohort of 88 patients with myeloid malignancies undergoing HSCT, correlating VDR polymorphisms of both recipients and donors with prognosis [178]. Like Cho et al., they detected a marked trend towards a decreased risk of acute GVHD in recipients with AA genotype (low VDR activity). Receptors with aa genotype and high VDR activity showed a trend toward a higher risk of acute GVHD, although differences were not statistically significant. However, recipients of donors with low VDR activity (AA) had a higher risk of death.

Bogunia-Kubik et al. published an analysis on 123 patients [177]. They found an association between the FokI FF genotype, which is associated with increased VDR activity and patient’s prognosis. If the donor and the recipient had the FF genotype, the recipients had a higher risk of GVHD. The Apal genotype, as in other studies, also had an impact on the risk of GVHD. Contrary to the data described in the study by Middleton et al., the AA donor genotype (low VDR activity) was associated with a higher risk of GVHD as compared to the genotype that had at least one an allele. At the same time, aa receptors (high VDR activity) had a higher risk of death and GVHD compared to the genotype with low VDR activity, which is consistent with other studies.

Thus, the vitamin D receptor and its mediation on immune signalling appear to have an impact on immune reconstitution after HSCT and the risk of infection and graft versus host disease.

Remarkably, a significant proportion of patients display low levels of vit D before HSCT [179,180]. In this regard, several studies have been reported describing the impact of the vitamin D levels before HSCT and post-transplant outcomes. More specifically, von Bahr et al. described an association between low levels of vit D and an increased risk of GVHD and CMV reactivation [180]. Similar results have been described by Hansson et al., who described an increased risk of death, relapse and cGVHD among patients with low vit D levels although, strikingly, grades 2 to 4 aGVHD occurred more frequently among patients with normal levels of Vitamin D [181]. Moreover, a higher risk of relapse has also been described among patients with low vitamin D levels. In this regard, Radujkovic et al. analysed a series of 492 patients undergoing HSCT; results were validated in an independent cohort of 398 patients. 396 (80%) and 348 (87%) patients had vit D deficiency before transplant in the training and validation cohort, respectively. Vit D deficiency was significantly associated with inferior overall survival, which was mainly attributed to a higher risk of relapse (HR, 1.96; *p* = 0.006) in patients diagnosed with myeloid (HR, 2.55; *p* = 0.014) but not with lymphoid malignancies (HR, 1.60; *p* = 0.147) [166].

With these data in mind we designed a phase I/II prospective trial in which 150 patients were included in three consecutive cohorts of 50 patients each group: control group (who did not receive vitamin D); low dose group (1000 UI vitamin D daily) and high dose group (5000 UI vitamin D daily). No significant differences were observed in terms of acute GVHD, relapse, non-relapse mortality and overall survival. By contrast, a significantly lower cumulative incidence of both overall and moderate plus severe chronic GVHD at 1 year was observed in patients receiving low (37.5% and 19.5%, respectively) or high doses of vitamin D (42.4% and 27%, respectively) as compared to the control group (67.5% and 44.7%, respectively) (*p* < 0.05). In multivariable analysis, treatment with vitamin D significantly decreased the risk of both overall (for low dose (HR = 0.31, *p* = 0.002) and for high dose of vitamin D (HR = 0.36, *p* = 0.006)) and moderate plus severe cGVHD (for low dose (HR = 0.22, *p* = 0.001) and for high dose vitamin D (HR = 0.33, *p* = 0.01)). There were no adverse events attributed to the vitamin D, more specifically, no case of hypercalcemia was observed. With this low toxicity profile, a prospective randomized trial would be required to confirm the potential efficacy of vitamin D as immune-modulatory agent after HSCT [182].

## 5. Conclusions

Our knowledge of vitamin D effects has grown in the past 20 years. The mechanisms of action and the role of vitamin D receptor in addition to its classic effects on calcium and bone homeostasis is well stablished. Vitamin D receptor is expressed on immune cells, which are all capable of synthesizing the active vitamin D metabolite. Moreover, vitamin D has the capability to act in an autocrine manner and can modulate the innate and adaptive immune responses.

As far as the clinical applications of vitamin D is concerned, several studies have been reported both in myeloid as well as in lymphoid malignancies suggesting that vitamin D may promote tumour cells differentiation and might play a role, in combination with other agents, for the treatment of these disorders, although no prospective randomized study is available to confirm these findings. In addition, in the transplant setting, the effect of vitamin D on the immune system might also influence patient’s outcome and, in this regard, different studies have evaluated the relationship between vitamin D levels pre-HSCT and risk of infections, graft-versus-host disease and relapse after transplantation. A phase I/II prospective trial suggests the potential benefit of the use of vitamin D to prevent GvHD. Prospective randomized trials would be required to confirm these findings.

## Abbreviations

vit D	Vitamin D
25(OH)D	Calcidiol, 25 hydroxycholecalciferol or 25-hidroxivitamin D
1,25(OH)_2_D3	1,25-Dihydroxyvitamin D3
DBP	Vitamin D-binding protein
CYP2R1	Vitamin D 25-hydroxylase or cytochrome P450 2R1
CYP27B1	1-α-Hydroxylase (1α-hydroxylase)
CYP24A1	Cytochrome P450 family 24 subfamily A member 1
FGF-23	Fibroblast growth factor 23
PTH	Parathyroid hormone
TNFα	Tumour necrosis factor α
IFNγ	Interferon gamma
UV	Ultra violet
VDR	Vitamin D receptor
RXR	Retinoid-X-receptor
VDRE	Vitamin D responsive elements
HAT	Histone acetyl transferases
NCoR	Nuclear receptor corepressor
SMRT NFAT	Retinoid or thyroid-hormone receptors Nuclear factor of activated T-cells
TSH	Thyroid-stimulating hormone
TRH	Thyrotropin-releasing hormone
TLR	Toll like receptor
NF-κB	Nuclear factor α B
DC	Dendritic cells
M-DCs	Myeloid dendritic cells
P-DCs	Plasmacytoid dendritic cells
MR	Manose receptors
CAMP	Cathelicidin
DEFB4	β defensin 2
CCR10	C-C chemokine receptor type 10
NOD2	Nucleotide-binding oligomerization domain-containing protein 2
SINE	Introduction of a nuclear element
PAMPs	Molecular patterns associated with pathogen surveillance
PRRs	Pattern recognition receptors
MDR	Multidrug resistance
HAMP	Hepcidin antimicrobial peptide
CFU-GM	Granulocyte macrophage colony forming cell
RAR	Retinoic acid receptor
RXR	Retinoid X receptor
SRC	Steroid receptor coactivator
DRIP	Vitamin D receptor interacting protein
PKC	Protein kinase C
MAPK	Mitogen activated kinase
iNKT	Invariant natural killer T cells
HSPC	Hematopoietic stem progenitor cell
HSC	Hematopoiectic stem cell
MDS	Myelodysplastic syndromes
AML	Acute myeloblastic leukaemias
ATRA	All-trans retinoic acid
APL	Acute promyelocytic leukaemia
EFS	Event free survival
AZA	5-Azacytidine
HR	Hazard ratio
CI	Confidence interval
OS	Overall survival
HSCT	Hematopoietic stem cell transplantation
CMV	Cytomegalovirus
cGVHD	Chronic graft-versus-host disease
aGVHD	Acute graft-versus-host disease
